# Utility of the American Association for the Surgery of Trauma Appendicitis Severity Grade in Predicting Prognosis in Children

**DOI:** 10.7759/cureus.65129

**Published:** 2024-07-22

**Authors:** Takayuki Fujii, Aya Tanaka, Hiroto Katami, Ryuichi Shimono

**Affiliations:** 1 Pediatric Surgery, Kagawa University, Takamatsu, JPN

**Keywords:** complicated appendicitis, anatomic severity, patient outcomes, grading system, acute appendicitis

## Abstract

Introduction: The American Association for the Surgery of Trauma (AAST) proposed a grade classification (I (mild) to V (severe)) to predict the risks and outcomes of acute appendicitis. However, its utility in children remains unknown. We investigated the relationship between the AAST grade and disease severity in children.

Methods: We retrospectively evaluated 93 patients aged ≤16 years who underwent surgery for acute appendicitis between 2012 and 2020. The AAST computed tomography (CT), operative, and pathologic grades were analyzed. We collected data for demographics, length of stay (LOS), and complications. Trend tests were performed to compare the AAST grade and outcomes. Bland-Altman plots were used to evaluate the correlation between grades. Multiple regression analysis was performed to identify the factors related to LOS.

Results: AAST grades included CT (n=55), operative (n=93), and pathologic (n=93) grades. The number of complications and LOS increased significantly with the increase of every three-grade. Bland-Altman plots revealed that each of the three-grade correlated with each other. Multiple regression analysis identified AAST operative grades III-V as risk factors for prolonged LOS.

Conclusion: Higher CT, operative, and pathologic grades were found to be significantly associated with an increased number of complications and prolonged LOS in pediatric patients. We further concluded that the AAST grading system could be useful in predicting the prognosis of acute appendicitis in children.

## Introduction

Although acute appendicitis is the most common surgical emergency in children, there are few indicators that can be used to objectively evaluate disease severity [1]. The classification of appendicitis as simple (non-perforated) or complex (gangrenous or perforated) appendicitis is widely used [2]. However, the clinical features of appendicitis vary significantly from patient to patient, and it is occasionally difficult to determine the exact condition of the disease using this dichotomous system [3]. Due to the lack of an accurate and standardized grading system for disease severity, a consensus among different hospitals, medical systems, or countries is difficult to reach [4].

Recently, the American Association for the Surgery of Trauma (AAST) proposed a grading system for acute appendicitis [5]. In this grading system, acute appendicitis is classified as grade Ⅰ (mild) to Ⅴ (severe) based on clinical, preoperative computed tomography (CT) imaging, intraoperative, and pathological findings [5]. In several studies of adult patients and two studies involving both adults and children, the AAST grade was found to be associated with the number of complications, length of stay (LOS), and cost [6-9]. Similarly, a recent retrospective cohort study of pediatric patients reported that the AAST grade was associated with increased complications and extended LOS [3]. However, this prior study only examined the intraoperative grade; as such, the association between each of the AAST grades and prognosis in children remains unclear.

Preoperative prediction of disease severity is important for keeping patients and their families informed, as well as in selecting the appropriate therapy. Pathological examination is also important in objectively confirming the clinical diagnosis of appendicitis and ensuring unusual diseases are not overlooked [10]. In addition, Collins et al. proposed that AAST grades should compare pediatric and adult patients separately, due to the correlation between age and grade [7]. In this context, in the present study, we investigated the relationship between disease severity with AAST, CT, operative, and pathologic grades in children.

## Materials and methods

Study patients

This was a retrospective, single-center cohort study. We evaluated children who underwent appendectomy for acute appendicitis in our hospital between April 2012 and October 2020. The exclusion criteria were as follows: patients aged ≥16 years old, those who only underwent drainage without appendectomy, or those who underwent interval appendectomy. We primarily administered piperacillin/tazobactam intravenously in patients who had perforated appendicitis or abscess formation. For other patients, cefmetazole was administered intravenously. Postoperative antibiotics were administered until the following criteria were achieved: reduced pain, body temperature <37.5°C for more than 24 hours, normal white blood cell (WBC) count, and lower C-reactive protein (CRP) level. In some cases, intravenous antibiotics were switched to oral antibiotics once these criteria were achieved. The criteria for discharge were the ability to take oral intake properly with no worsening of symptoms for ≥24 hours after intravenous antibiotic treatment was completed, or after switching to oral antibiotics. This study was approved by the ethics committee of the Faculty of Medicine of Kagawa University (registration number: 2019-257). The need for informed consent was waived due to the retrospective and observational nature of the study.

Data collection

The AAST CT, operative, and pathologic grades were analyzed [5]. The descriptions of AAST grade for acute appendicitis were as follows: grade I: acutely inflamed appendix, intact; grade II: gangrenous appendix, intact; grade III: perforated appendix with local contamination; grade IV: perforated appendix with periappendiceal phlegmon or abscess; grade V: perforated appendix with generalized peritonitis [5]. The lead author (T.F.) performed all grading. However, when it was difficult to assign a grade, the lead author consulted with another researcher (A.T.) to increase validity. All data were collected from the electronic medical records at our hospital. The CT grade was assigned from the CT report, the operative grade was assigned from the operative note, and the pathologic grade was assigned from the pathology report. In cases where it was difficult to determine the grade based solely on the radiology report or operative note, the CT images or surgery videos were assessed. As baseline demographic data, we examined the age, sex, and type of surgical procedure. We further compared the correlation between grades and age, body mass index (BMI), body temperature, WBC, CRP level, time from onset to surgery, and surgery time. To assess the severity of acute appendicitis, we compared the number of complications within 30 days of surgery and LOS.

Statistical analysis

A trend test was conducted to assess the association between grades using the Jonckheere-Terpstra test (continuous variables) or Cochran-Armitage test (categorical variables) with Eazy R (Saitama Medical Center, Jichi Medical University, Saitama, Japan) [11]. Bland-Altman plots were applied to evaluate the correlation between CT, operative, and pathologic grades using MEDCALC (MedCalc Software Ltd., Ostend, Belgium). Stepwise multiple regression analysis was further performed using IBM SPSS Statistics (SPSS Inc., Chicago, IL) to identify factors associated with LOS. Based on previous studies, AAST operative grades II-V, body temperature ≥38°C, WBC >16000, and >3 days from onset to surgery were included in the analysis [3]. Statistical significance was set at p<0.05.

## Results

Of the 112 patients who underwent appendectomy for acute appendicitis in our hospital between April 2012 and October 2020, 19 were excluded because they underwent interval appendectomy. Consequently, 93 patients were enrolled in this study. The CT grade was evaluated in 55 patients who underwent CT examination. The patients had a median age of 9.8 years (interquartile range: 7.8-12.3), and 42% were female. Only three patients underwent open appendectomy, while the rest underwent laparoscopic appendectomy. Table 1 shows the patient characteristics and outcomes based on AAST CT grade (n=55; Ⅰ, 27; Ⅱ, 10; Ⅲ, 5; Ⅳ, 5; and Ⅴ, 8).

**Table 1:** Patient characteristics and outcomes based on AAST CT grade Data are presented as the median (interquartile range) or number (%). ^a^Jonckheere-Terpstra test was done. ^b^Cochran-Armitage test was done. Reference range: CRP (0-0.14 mg/dL), WBC (3.3-8.6×1000 /μL). AAST, American Association for the Surgery of Trauma; BMI, body mass index; CT, computed tomography; SSI, surgical site infection, WBC, white blood cell count; LOS, length of stay; CRP, C-reactive protein

	AAST CT grade (n=55)					
Variable	Ⅰ	Ⅱ	Ⅲ	Ⅳ	Ⅴ	p-value
Patients, n (%)	27 (49)	10 (18)	5 (9)	5 (9)	8 (15)	
Age (years)	12 (9.1-14)	8.5 (7.6-10)	9.4 (4.7-12)	9.7 (7.5-10)	7.8 (5.7-9.7)	0.002^a^
BMI (kg/m^2^)	18 (15-21)	16 (15-17]	16 (14-17)	16 (16-16)	16 (14-18)	0.084^a^
Temperature (°C)	37.4 (36.8-38.1)	37.3 (37.1-37.7)	37.8 (37.5-38.2)	38.0 (37.9-39.0)	38.4 (38.3-39.2)	0.017^a^
WBC×1000 (/μL)	15.4 (13.7-18.1)	16.0 (11.1-20.9)	16.2 (14.6-16.5)	20.5 (16.2-22.0)	16.2 (10.1-19.4)	0.49^a^
CRP (mg/dL)	1.0 (0.2-4.4)	3.7 (0.9-8.0)	4.3 (1.5-6.7)	9.2 (6.6-9.2)	10.3 (9.0-20.2)	<0.001^a^
Time from onset to surgery (day)	1.0 (1.0-2.0)	2.0 (1.0-2.0)	2.0 (1.0-2.0)	3.0 (2.0-3.0)	2.0 (2.0-3.0)	<0.001^a^
Surgery time (min)	91 (77-116)	94 (78-109)	92 (87-122)	143 (139-149)	136 (104-154)	0.004^a^
Complications, n (%)	1 (4)	1 (10)	0 (0)	2 (40)	3 (38)	0.004^b^
Superficial SSI, n (%)	1 (4)	1 (10)	0 (0)	0 (0)	1 (13)	
Organ space SSI, n (%)	0 (0)	0 (0)	0 (0)	2 (40)	1 (13)	
Ileus, n (%)	0 (0)	0 (0)	0 (0)	0 (0)	1 (13)	
Postlaparoscopic shoulder pain, n (%)	0 (0)	0 (0)	0 (0)	0 (0)	0 (0)	
LOS (days)	8.0 (7.0-9.5)	7.5 (7.0-8.0)	10 (9.0-11)	13 (9.0-15)	12 (9.0-13)	0.002^a^

Younger age, higher body temperatures, and elevated CRP levels were all associated with higher grades. Seven cases experienced complications including superficial surgical site infections (SSI) (n=3), organ space SSI (n=3), and ileus (n=1). The number of complications and LOS increased significantly with increasing grade (p=0.004 and p=0.002, respectively). Table 2 presents the patient characteristics and outcomes based on AAST operative grade (n=93; Ⅰ, 46; Ⅱ, 17; Ⅲ, 10; Ⅳ, 10; and Ⅴ, 10).

**Table 2:** Patient characteristics and outcomes based on AAST operative grade Data are presented as the median (interquartile range) or number (%). ^a^Jonckheere-Terpstra test was done. ^b^Cochran-Armitage test was done. Reference range: CRP (0-0.14 mg/dL), WBC (3.3-8.6×1000 /μL). AAST, American Association for the Surgery of Trauma; BMI, body mass index; CRP, C-reactive protein; SSI, surgical site infection; WBC, white blood cell; LOS, length of stay

	AAST operative grade (n=93)					
Variable	Ⅰ	Ⅱ	Ⅲ	Ⅳ	Ⅴ	p-value
Patients, n (%)	46 (49)	17 (18)	10 (11)	10 (11)	10 (11)	
Age (years)	11 (8.2-13)	9.8 (6.1-12)	8.2 (5.1-9.0)	10 (8.5-12)	9.4 (6.3-10)	0.03^a^
BMI (kg/m^2^)	16 (15-19)	16 (15-18)	16 (15-16)	18 (14-20)	16 (15-18)	0.48^a^
Temperature (°C)	37.2 (36.7-37.6)	37.5 (36.7-38.2)	38.4 (38.2-38.6)	38.2 (37.3-39.2)	38.4 (38.2-39.5)	<0.001^a^
WBC×1000 (/μL)	14.2 (12.0-18.0)	17.6 (14.5-18.3)	19.5 (15.7-22.6)	19.8 (15.6-21.8)	16.2 (11.3-19.7)	0.008^a^
CRP (mg/dL)	0.8 (0.2-2.3)	3.7 (1.3-5.9)	6.2 (3.8-10)	9.2 (7.7-14)	10 (8.8-23)	<0.001^a^
Time from onset to surgery (day)	1.0 (1.0-2.0)	1.0 (1.0-2.0)	2.0 (2.0-2.0)	3.0 (2.3-3.0)	2.0 (2.0-3.0)	<0.001^a^
Surgery time (min)	81 (73-99)	102 (86-123)	121 (106-126)	141 (129-157)	137 (111-149)	<0.001^a^
Complications, n (%)	3 (6.5)	1 (5.9)	1 (10)	2 (20)	4 (40)	0.005^b^
Superficial SSI, n (%)	1 (2.2)	1 (5.9)	0 (0)	0 (0)	1 (10)	
Organ space SSI, n (%)	0 (0)	0 (0)	1 (10)	2 (20)	1 (10)	
Ileus, n (%)	1 (2.2)	0 (0)	0 (0)	0 (0)	2 (20)	
Postlaparoscopic shoulder pain, n (%)	1 (2.2)	0 (0)	0 (0)	0 (0)	0 (0)	
LOS (days)	7.5 (7.0-8.0)	8.0 (7.0-9.0)	10 (9.3-12)	11 (9.0-14)	12 (9.3-13)	<0.001^a^

Younger age, higher body temperatures, and elevated CRP levels and WBC were all associated with higher grades. Eleven cases experienced complications, including superficial SSI (n=3), organ space SSI (n=4), ileus (n=3), and postlaparoscopic shoulder pain (n=1). The number of complications and LOS increased with increasing grade (p=0.005 and p<0.001, respectively). Table 3 presents the patient characteristics and outcomes based on AAST pathologic grade (n=93; Ⅰ, 41; Ⅱ, 24; Ⅲ, 9; Ⅳ, 10; and Ⅴ, 9).

**Table 3:** Patient characteristics and outcomes based on AAST pathologic grade Data are presented as the median (interquartile range) or number (%). ^a^Jonckheere-Terpstra test was done. ^b^Cochran-Armitage test was done. Reference range: CRP (0-0.14 mg/dL), WBC (3.3-8.6×1000 /μL). AAST, American Association for the Surgery of Trauma; BMI, body mass index; CRP, C-reactive protein; SSI, surgical site infection; WBC, white blood cell; LOS, length of stay

	AAST pathologic grade (n=93)					
Variable	Ⅰ	Ⅱ	Ⅲ	Ⅳ	Ⅴ	p-value
Patients, n (%)	41 (44)	24 (26)	9 (10)	10 (11)	9 (10)	
Age (years)	11 (7.9-12)	11 (8.1-13)	7.8 (4.7-8.6)	10 (8.5-12)	9.3 (6.2-10)	0.10^a^
BMI (kg/m^2^)	16 (15-19)	16 (15-17)	14 (14-16)	18 (16-20)	17 (15-18)	0.64^a^
Temperature (°C)	37.0 (36.7-37.7)	37.4 (37.1-37.9)	38.3 (38.2-38.5)	38.2 (37.3-39.2)	38.5 (38.3-39.6)	<0.001^a^
WBC×1000 (/μL)	14.0 (11.7-16.2)	18.0 (14.5-20.9)	16.8 (15.4-22.6)	19.8 (15.6-21.8)	16.7 (13.2-19.9)	<0.001^a^
CRP (mg/dL)	0.8 (0.1-2.4)	3.9 (1.2-7.4)	5.7 (2.5-9.6)	9.2 (7.7-14)	11 (9.7-24)	<0.001^a^
Time from onset to surgery (day)	1.0 (1.0-2.0)	1.0 (1.0-2.0)	2.0 (2.0-2.0)	3.0 (2.3-3.0)	2.0 (2.0-3.0)	<0.001^a^
Surgery time (min)	80 (72-104)	92 (81-107)	122 (120-127)	141 (129-157)	140 (105-150)	<0.001^a^
Complications, n (%)	3 (7.3)	1 (4.2)	2 (22)	2 (20)	3 (33)	0.015^b^
superficial SSI (n)	1 (2.4)	1 (4.2)	0 (0)	0 (0)	1 (11)	
organ space SSI (n)	0 (0)	0 (0)	1 (11)	2 (20)	1 (11)	
Ileus (n)	1 (2.4)	0 (0)	1 (11)	0 (0)	1 (11)	
Postlaparoscopic shoulder pain (n)	1 (2.4)	0 (0)	0 (0)	0 (0)	0 (0)	
LOS (days)	7.0 (7.0-9.0)	8.0 (7.0-8.3)	10 (10-12)	11 (9.0-14)	12 (9.0-13)	<0.001^ a^

Higher body temperatures and elevated CRP and WBC levels were associated with higher grades. The number of complications and LOS increased with increasing grade (p=0.015 and p<0.001, respectively).

**Figure 1:**
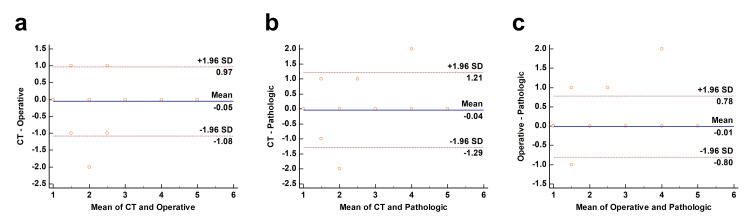
Bland-Altman analysis comparing each AAST grade (a) CT and operative grade (n=55). (b) CT and pathologic grade (n=55). (c) Operative and pathologic grade (n=93). The blue line indicates the bias (mean of difference). The red line shows the 95% limits of agreement (two standard deviations of difference). AAST, American Association for the Surgery of Trauma; CT, computed tomography

Figure 1 shows the Bland-Altman plots between the CT and operative (Figure 1a; n=55) and pathologic grades (Figure 1b; n=55), as well as between operative and pathologic grades (Figure 1c; n=93).

The correlation of CT and operative grades with a 95% confidence interval for the mean difference was -0.20 to 0.087 (p=0.44), with a coefficient of repeatability of 1.0. The correlation of CT and pathologic grades with a 95% confidence interval for the mean difference was -0.21 to 0.14 (p=0.67), with a coefficient of repeatability of 1.2. The correlation of operative and pathologic grades with a 95% confidence interval for the mean difference ranged from -0.094 to 0.072 (p=0.80), with a coefficient of repeatability of 0.8. In all comparisons, there was no evidence of systematic bias, such as fixed or proportional bias.

Multiple regression analysis was further performed to detect factors associated with LOS. There was no variable for which | r |> 0.9. Using the Shapiro-Wilk test, the LOS was confirmed to be non-normally distributed, while analysis was performed using 1/LOS as the variable transformation (Table 4).

**Table 4:** Multiple regression analysis for variables decreasing 1/LOS R^2^=0.34; ANOVA, p<0.001. Stepwise multiple regression analysis was performed. LOS, length of stay

Variable	B	β	p-value	95% confidence interval
constant	0.13		0.0	0.12～0.14
Operative grade III	-0.028	-0.28	0.0	-0.053～-0.017
Operative grade Ⅳ	-0.044	-0.41	0.0	-0.058～-0.021
Operative grade Ⅴ	-0.053	-0.54	0.0	-0.064～-0.028

ANOVA results were statistically significant (p<0.001), with an R^2^ of 0.34. The Durbin-Watson ratio was 2.0. We further identified AAST operative grades III-V as risk factors for prolonged LOS.

## Discussion

The present study investigated the relationship between AAST grade and LOS and the number of complications as an indicator of acute appendicitis severity in children. We further investigated the correlation between CT, operative, and pathologic grades, as well as factors associated with LOS with respect to operative grade. Our study further revealed that LOS and the number of complications increased significantly with increasing CT, operative, and pathologic grades in children. Furthermore, Bland-Altman plots revealed that each of the three grades correlated with each other. We identified AAST operative grades III-V as risk factors for prolonged LOS.

Recently, the AAST proposed a grading system that classified acute appendicitis from grades I (mild) to V (severe) [5]. In several studies enrolling adults, the AAST grading system was proven to be a valid predictor of complications, LOS, and cost [6-8,12]. Overall, these findings indicate that the AAST grading system could be useful in determining treatment strategies for acute appendicitis. In 2018, the AAST proposed recommendations for the treatment of acute appendicitis stratified by AAST grade, based on published consensus guidelines [13]. Furthermore, one multi-center retrospective case-control study confirmed that AAST CT grade seems to show a relevant prognostic value and a potential impact on the choice of surgical strategy [14].

In addition to previous studies including adults, the present study revealed that complications and LOS increased with higher CT, operative, and pathologic grades [6-8]. Similarly, one study of AAST operative grades in children revealed that complications were increased and that LOS was extended with increasing grades [3]. These findings indicate that AAST grades could be useful in predicting appendicitis prognoses in pediatric patients as well as adults.

The values of the coefficient of repeatability in this study (0.8-1.2) did not differ significantly from previously reported values (0.7-0.9) [6,7]. Furthermore, the values of this study were clinically acceptable and appeared to be compatible with each grade. The correlation between imaging and operative findings and pathology results enables the scientific determination of the patient's subsequent treatment strategy based on CT or operative grades, even before the pathology results are available.

Ultrasonography (US) is recommended as the first-line diagnostic imaging modality for appendicitis in children due to its low cost, avoidance of sedation, contrast agents, and minimal radiation exposure [15]. Although US imaging criteria are not included in the AAST grading system, Collins et al. examined imaging grades using the US and reported the lowest correlation between other grades (clinical, CT, operative, and pathologic grades) [7]. Although the US depends on the skill of the operator and the physique of the patients, further investigation is required to determine how the US can replace the AAST CT grade in order to reduce radiation exposure in children.

The AAST grades are expected to become a useful research platform for the objective and detailed evaluation of patients with acute appendicitis. Hernandes previously advocated that the AAST grading system is necessary for clinical practice and research of patients with acute appendicitis, as it can reliably assign disease severity and predict patient outcomes, while providing a robust universal classification system with high inter-rater reliability [16]. Indeed, one study using the AAST grade showed that rural residence was associated with anatomical severity and poorer outcomes of appendicitis in pediatric patients [17].

The incidence of shoulder pain following laparoscopic appendectomy has been reported to be approximately 7-32% [18,19]. Although the exact mechanism underlying the development of shoulder pain after laparoscopic surgery has yet to be elucidated, the prevailing hypothesis is that the pneumoperitoneum pressure causes excessive stretching of the diaphragm, resulting in referred pain to the shoulder via the phrenic nerve [20]. In the present study, we encountered only one case of shoulder pain, representing a lower incidence than that observed in previous studies. However, this patient complained of severe shoulder pain. Furthermore, it should be noted that, due to the nature of retrospective studies such as this, even if other patients had slight shoulder pain, if they did not complain about it, it may not have been recorded in the medical record.

This study has several limitations. First, due to the retrospective nature of the study, it was occasionally difficult to assign a grade based solely on medical charts. Furthermore, the AAST grades were predominantly assigned by a single researcher; however, if it was difficult to assign the grade, this researcher had a conversation with another researcher to increase validity. Second, the number of cases was small, which may have weakened the significance of our findings.

To resolve these problems, a prospective study with a larger number of subjects should be performed in the future. Despite these limitations, to our knowledge, this is the first study to evaluate AAST CT and pathological grade in children.

## Conclusions

Higher CT, operative, and pathologic grades were found to be significantly associated with an increased number of complications and prolonged LOS in pediatric patients. We further concluded that the AAST grading system could be useful in predicting the prognosis of acute appendicitis in children. Our findings could further assist in selecting more appropriate treatment strategies and improving the outcomes of these patients.
